# miRNAs involved in the regulation of exercise fatigue

**DOI:** 10.3389/fphys.2025.1614942

**Published:** 2025-06-23

**Authors:** Siyuan Niu, Xiupeng Yin, Qinglei Cao, Kaiyu Huang, Zhongyuan Deng, Jie Cao

**Affiliations:** ^1^ College of Physical, Sungshin University, Seoul, Republic of Korea; ^2^ College of Science and Technology, Zhengzhou University, Zhengzhou, China; ^3^ Department of Physical Education, University of Science and Technology Beijing, Beijing, China; ^4^ School of Agriculture, Zhengzhou University, Zhengzhou, China; ^5^ College of Physical, Kookmin University, Seoul, Republic of Korea

**Keywords:** exercise fatigue, microRNA, energy metabolism, inflammatory response, oxidative stress

## Abstract

Exercise-induced fatigue refers to a temporary decline in physiological function resulting from prolonged or high-intensity exercise, which is characterized by decreased muscle strength, diminished exercise performance, and heightened subjective feelings of fatigue. The study of exercise fatigue holds significant importance not only in competitive sports and public health, but also extends to medicine, military applications, and occupational safety. MicroRNA (miRNA) represents a class of non-coding RNA that plays a pivotal role in the regulation of gene expression. The involvement of miRNAs in exercise-induced fatigue has garnered increasing attention within the scientific community. This article provides an overview of fundamental concepts and biological functions associated with miRNAs, defines and classifies exercise fatigue while outlining its physiological changes, emphasizes alterations in miRNA expression during episodes of exercise-induced fatigue, and conducts an in-depth analysis regarding the mechanisms through which miRNAs influence this phenomenon via modulation of energy metabolism, inflammatory responses, and oxidative stress. Furthermore, this article anticipates future research directions as well as potential clinical applications for miRNAs concerning exercise-induced fatigue. This review holds significant importance for elucidating the molecular mechanisms underlying exercise-related fatigue while fostering advancements within sports medicine and rehabilitation science.

## 1 Introduction

Exercise-induced fatigue typically refers to the phenomenon of reduced exercise capacity resulting from a temporary imbalance in physiological functions during continuous or high-intensity physical activity (Gandevia, 2001). It is characterized by decreased contractility of skeletal muscles, decreased efficiency of neural drive, and increased subjective fatigue perception. Exercise fatigue can be categorized into peripheral fatigue (such as dysfunction within the muscular system) and central fatigue (such as abnormal regulation by the nervous system), based on its location. These two forms of fatigue interact dynamically through the neuromuscular coupling system (Carroll et al., 2017). There are several factors contributing to the phenomenon of exercise-induced fatigue. The most prominent reasons include an imbalance between the resynthesis rate and consumption rate of adenosine triphosphate (ATP), disturbances in the internal environment of muscle cells resulting from high-intensity exercise, dynamic fluctuations in neurotransmitter levels within pathways involving the prefrontal cortex, basal ganglia, and cerebellum, as well as heat stress (Renaud et al., 2023).

MicroRNAs (miRNAs) are a class of endogenous small non-coding RNA molecules, typically ranging from 18 to 24 nucleotides in length. In 1993, researchers first identified the microRNA lin-4 in the nematode *Caenorhabditis elegans*, which regulates larval development by inhibiting the translation of its target gene lin-14 through incomplete base pairing (Lee et al., 1993). In 2000, a second microRNA, let-7, was discovered; it also plays a crucial role in regulating developmental timing in nematodes and has been shown to be highly conserved across different species (Reinhart et al., 2000). The 2024 nobel prize in physiology or medicine has been awarded jointly to Victor Ambros and Gary Ruvkun for their groundbreaking discovery of microRNAs—small non-coding RNA molecules that regulate gene expression. Their seminal work, beginning with the identification of lin-4 (Lee et al., 1993) and let-7 (Reinhart et al., 2000) in *C. elegans*, revealed an entirely new layer of genetic regulation conserved across species. This paradigm-shifting finding not only transformed our understanding of developmental timing and cellular communication but also opened new avenues for diagnosing and treating diseases, including cancer and neurological disorders. In 2001, researchers formally introduced the term “microRNA” and predicted the existence of hundreds of such molecules in animals (Lagos-Quintana et al., 2001). By 2002, it was established that microRNAs mediate target mRNA degradation or translational repression via the RNA-induced silencing complex (RISC) (Hutvágner and Zamore, 2002). In 2005, the miRBase database was launched as an authoritative resource for miRNA annotation. In the past decade, microRNAs (miRNAs) have emerged as significant regulators of gene expression and have gradually gained recognition in the field of exercise physiology. During physical activity, miRNAs influence various cellular metabolic pathways, including energy acquisition, inflammation, oxidative stress, and numerous other cellular processes. Additionally, they play crucial roles in cell proliferation, development, and anti-apoptotic mechanisms. These diverse functions underscore the importance of miRNAs as essential molecules for understanding exercise-induced fatigue (Safdar et al., 2016).

Previous studies have established that microRNAs (miRNAs) play a significant role in the onset and progression of exercise-induced fatigue. The alterations in the expression levels of miRNA-1 and miRNA-21 following exercise fatigue are negatively correlated with muscle damage and repair (Meeusen et al., 2021). Specifically, miR-23a may exacerbate exercise-induced fatigue by targeting PGC-1α, thereby inhibiting mitochondrial biosynthesis (Wada et al., 2011). Conversely, miR-146a mitigates inflammation by suppressing TRAF6/NF-κB signaling pathways post-exercise (Baggish et al., 2011). Furthermore, miRNAs are frequently utilized as biomarkers for assessing exercise fatigue (Guescini et al., 2015; Bye et al., 2013). Collectively, these findings suggest that miRNAs represent key molecular players in elucidating the mechanisms underlying exercise fatigue through their regulation of muscle repair, energy metabolism, and inflammatory responses (Baek et al., 2024).

Exercise fatigue is a multi-dimensional and complex phenomenon that involves physiological, metabolic, and nervous systems. As key regulators of gene expression, miRNAs target genes associated with muscle repair, energy metabolism, inflammatory responses, and central nervous system function. They elucidate their regulatory networks at various levels, thereby contributing to the construction of a more comprehensive molecular mechanism regulatory network. This article reviews the research advancements concerning miRNAs in exercise fatigue. Such insights not only enhance our understanding of exercise fatigue but also provide theoretical support and technical groundwork for fields such as sports medicine, rehabilitation science, and the health industry. Looking ahead, with the progression of precision medicine and molecular diagnostic technologies, miRNA-based monitoring and intervention strategies for fatigue are anticipated to emerge as one of the core research directions within sports science.

## 2 Basic concepts and biological functions of miRNA

**FIGURE 1:**
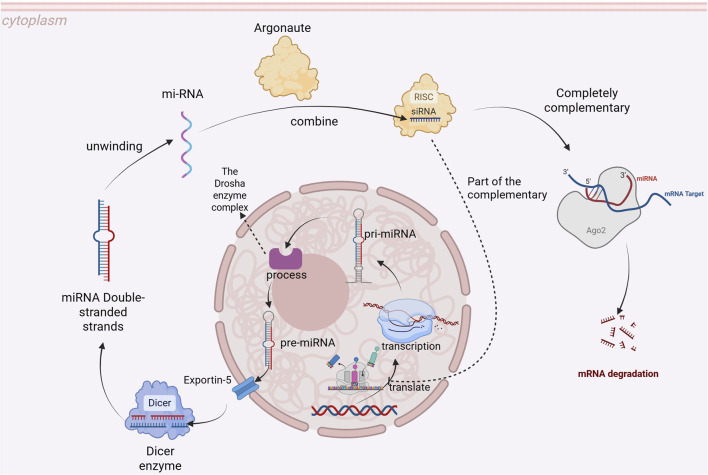
Process of miRNA Formation and Its Mechanism of Action.

MicroRNAs are widely distributed across both plant and animal kingdoms and play crucial roles in various biological processes by regulating gene expression. miRNAs can be categorized based on their genomic location into intergenic miRNA and intragenic miRNA, as well as based on evolutionary conservation into conserved miRNA and non-conserved miRNA. The genes encoding miRNAs are transcribed by RNA polymerase II to produce primary miRNAs (pri-miRNAs), which are subsequently processed by the Drosha enzyme complex into precursor miRNAs (pre-miRNAs). These pre-miRNAs are then transported to the cytoplasm via Exportin-5, where they undergo cleavage by Dicer enzymes to generate mature miRNA duplexes. The resulting double-stranded mature miRNA unwinds, with one strand—known as the guide strand—binding to Argonaute proteins to form the RNA-induced silencing complex (RISC). miRNAs induce mRNA degradation when there is full complementarity with their target mRNA, while partial complementarity leads to inhibition of mRNA translation (Figure 1). Numerous studies have demonstrated that miRNAs participate in diverse cellular activities such as cell proliferation, differentiation, and programmed cell death. Additionally, they play significant roles in various disease-related pathophysiological processes including cancer, cardiovascular diseases, metabolic disorders (Ambros, 2004; Bartel, 2004; He and Hannon, 2004; Falabrègue et al., 2021; Ma et al., 2021).

The miRNAs play a crucial role in the regulation of various cellular processes, including proliferation, apoptosis, differentiation, metabolism, immune response, stress response, migration and invasion, as well as aging (Figure 1). miR-21 enhances cell proliferation and inhibits apoptosis by downregulating the expression of the tumor suppressor gene PTEN (Esquela-Kerscher and Slack, 2006). Conversely, the miR-34 family promotes apoptosis through modulation of the p53 pathway, thereby inhibiting tumor growth (Garzon et al., 2010). Moreover, miR-145 is involved in smooth muscle cell differentiation while miR-133 regulates cardiomyocyte differentiation. Additionally, both miR-1 and miR-133 are essential for skeletal muscle differentiation (Chen et al., 2006; Ivey and Srivastava, 2010). The miR-33 family influences cholesterol metabolism by regulating sterol regulatory element-binding protein (SREBP). Notably, miR-122 is highly expressed in the liver and participates in lipid metabolism and cholesterol synthesis regulation (Rayner et al., 2010; Rottiers and Näär, 2012). Furthermore, miR-155 plays a significant role in B cell and T cell immune responses. miR-146a mitigates inflammatory responses by modulating the NF-kB pathway (Taganov et al., 2006; O’Connell et al., 2007). miR-210 is implicated in hypoxic conditions through its regulation of the HIF-1α pathway (Crosby et al., 2009). Similarly, miR-34a promotes cellular apoptosis following DNA damage via its influence on the p53 pathway (Kulshreshtha et al., 2007). The members of the miR-200 family inhibit tumor cell migration and invasion by obstructing epithelial-mesenchymal transition (EMT) through suppression of ZEB1/2 expression (Gregory et al., 2008). Conversely, miR-9 enhances tumor metastasis by regulating E-cadherin expression levels (Ma et al., 2010). Furthermore, miR-34a facilitates cellular senescence via SIRT1 modulation while the miR17–92 cluster delays senescence through inhibition of p21 (Boehm and Slack, 2005; Bhaumik et al., 2009). In summary, miRNAs play a crucial role in various cellular physiological processes by regulating gene expression. Future research is expected to further elucidate the role of miRNAs in the onset and progression of diseases and to provide new targets for disease diagnosis and treatment.

## 3 Physiological mechanisms of exercise fatigue

### 3.1 Definition and classification of exercise fatigue

Exercise fatigue is typically defined as a temporary decline in physical function following engagement in sports activities, which results in diminished athletic performance. This form of fatigue can manifest as either physical or psychological. Physical exercise fatigue arises from the depletion of muscle energy reserves, the accumulation of metabolites (such as lactic acid), and a reduction in nerve conduction efficiency. In contrast, psychological exercise fatigue is associated with various psychological factors including emotion, attention, and motivation. Based on different classification criteria for exercise fatigue, it can be categorized into acute exercise fatigue and chronic exercise fatigue (Table 1). Acute exercise fatigue refers to the sensation of tiredness experienced immediately after a single bout of high-intensity exercise; this type of fatigue generally resolves within a short period. Conversely, chronic exercise fatigue denotes persistent exhaustion resulting from prolonged high-intensity training or activity, characterized by an extended recovery time and potentially accompanied by other health issues (Lehninger et al., 2005). Furthermore, according to the site of origin, exercise fatigue can also be classified into central exercise fatigue (Table 1) (originating from the central nervous system) and peripheral exercise fatigue (stemming from muscle tissue). There are notable differences in physiological mechanisms between these two types of exercise-induced fatigues. The following is a comprehensive summary of the classification and characteristics associated with exercise-induced fatigue.

**TABLE 1:** Comparison of key characteristics of different types of exercise fatigue.

Type	Definition	Reason	Recovery time	Other characteristics
Physiological fatigue	Fatigue caused by psychological factors such as emotion, attention and motivation	Long-term mental stress, insufficient psychological recovery, lack of motivation, emotional instability and so on	The recovery time is long and may require psychological intervention	It affects performance, decision-making ability, emotional state and so on
Acute fatigue	Fatigue occurring immediately after a high-intensity exercise	Muscle energy reserve consumption, accumulation of metabolites (such as lactic acid), and reduced nerve conduction efficiency	Usually recovered in a short time	Mainly temporary and does not lead to long-term health problems
Chronic fatigue	Persistent fatigue caused by long-term intensive training or exercise	Long-term high-intensity exercise, over-training, insufficient recovery, etc	A long recovery time may be accompanied by health problems	It may be associated with other health problems (such as decreased immune function, anxiety, etc.)
Central fatigue	Fatigue originating from the central nervous system	Insufficient regulation of the nervous system, resulting in decreased motor performance	Recovery time depends on the specific cause	Affect motor performance, mood, attention, and motivation
Peripheral fatigue	Fatigue originating from the muscle tissue	Muscle energy expenditure, lactate accumulation, metabolites	Short recovery time, depending on the exercise intensity	Mainly affecting muscle strength and endurance

### 3.2 Physiological changes of exercise fatigue

Exercise fatigue is a phenomenon characterized by a decline in bodily function following prolonged or high-intensity exercise. The physiological changes associated with this condition involve multiple systems, including interactions among the nervous system, muscular system, and endocrine system (Table 2). During physical activity, energy depletion serves as the primary factor contributing to exercise fatigue; the muscle’s energy supply predominantly relies on the oxidation of glycogen and fat. When exercise fatigue manifests, it primarily presents as a reduction in muscle glycogen levels and ATP consumption, which leads to decreased muscle contractility (Bergström et al., 1967; Fitts, 1994). Additionally, the accumulation of metabolites such as lactate and hydrogen ions can lower muscle pH levels and disrupt both muscle contraction and neural signaling (Sahlin et al., 1998; McKenna et al., 2008). Furthermore, diminished neuromuscular function—including central nervous system fatigue and reduced muscle excitability—further compromises motor performance (Gandevia, 2001). Electrolyte imbalances, particularly losses of potassium and sodium ions, also adversely affect both muscular and neurological functions (Maughan et al., 2007). Concurrently, exercise-induced oxidative stress results in an increase in free radicals while weakening antioxidant defenses that are crucial for maintaining cellular structure and function (Ji, 1995; Powers and Jackson, 2008). Finally, hormonal fluctuations—such as elevated cortisol levels coupled with decreased testosterone—may suppress immune responses as well as hinder muscle repair processes (Ji, 1995). The cumulative effects of these physiological alterations ultimately lead to exercise fatigue that negatively impacts athletic performance and recovery capacity. A comprehensive understanding of the physiological mechanisms underlying exercise fatigue is not only advantageous for athlete training and rehabilitation but also holds significant implications for health management within the general population. The following is a concise overview of the physiological mechanisms underlying exercise-induced fatigue.

**TABLE 2:** A list of physiological changes, impacts, and mechanisms of various systems during exercise fatigue.

System	Physiological change	Influence	Related mechanism
Musculation	1. Source of energy: the oxidation of glycogen and fat 2. Glycogen consumption is accelerated, and lactate accumulates	Lactate accumulation causes a decrease in muscle contractility, causing pain and discomfort	Lactate accumulation acidifies the muscle, inhibiting the ability of the muscle to contract
Nervous system	1. The decline in the nerve conduction rate 2. Lower muscle excitability	Motor performance decreased, and motor coordination ability decreased	Prolonged exercise causes functional fatigue of the nervous system and reduces nerve conduction and muscle response speed
Endocrine system	Stress causes elevated cortisol levels	Increase in the body’s stress response, may affect the immune system and the ability to recover	High-intensity exercise causes increased secretion of stress hormones that may weaken the body’s antioxidant capacity
Antioxidant system	High-intensity exercise reduces the antioxidant capacity	Increase in oxidative stress capacity, affecting tissue repair and regeneration capacity	Increasing oxidative stress after exercise and decreased oxidative antioxidant capacity, affecting cell repair and regeneration

### 3.3 Changes in miRNA expression during exercise fatigue

**FIGURE 2:**
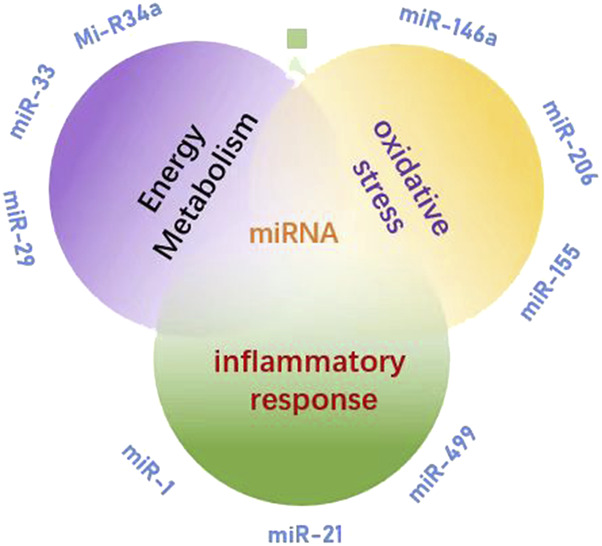
miRNA is involved in the research direction of exercise fatigue mechanism.

Exercise-induced alterations in miRNA expression profiles represent a multivariate dynamic process that encompasses various physiological and biochemical activities. Following exercise, the levels of certain miRNAs within the body undergo significant changes, which are closely associated with energy metabolism, inflammatory responses, oxidative stress, and muscle repair. Investigations into changes in miRNA expression during exercise fatigue underscore their critical role in regulating physiological adaptation and recovery post-exercise (Figure 2). Research has demonstrated that exosomal miRNAs circulating in the bloodstream exhibit substantial modifications following endurance training. For instance, muscle-specific miRNAs such as miR-1, miR-133, and miR-206 are upregulated after exercise and play pivotal roles in modulating muscle differentiation and repair (Hackney, 2006). Additionally, the expressions of both miR-21 and miR-146a increase post-exercise; these changes may be linked to the regulation of inflammatory responses and could aid in mitigating tissue damage resulting from physical activity (Yuasa et al., 2008). Conversely, variations in the expression of miR-23a and miR-486 correlate with oxidative stress and energy metabolism. Exercise intervention has significant tissue specificity for miRNA regulation in adipose tissue. A study in obese women showed that 12 weeks of combined aerobic resistance combination training significantly upregulated the expression of miR-155-5p and miR-329-3p in subcutaneous adipose tissue (GSAT), but not in abdominal subcutaneous fat (ASAT) (Carmen et al., 2022). These specific miRNAs influence mitochondrial function and antioxidant defense mechanisms by regulating target gene expression (Nielsen et al., 2014). Importantly, different types of exercise—such as aerobic versus resistance training—and varying intensities can lead to distinct differences in miRNA expression profiles. This suggests that the regulatory functions of miRNAs concerning exercise fatigue are complex and context-dependent (Russell et al., 2013). Moreover, it is crucial to recognize that the expression of miRNA is influenced by both the level and timing of physical exertion. Of concern is that exercise-induced exosomal miRNA has emerged as a new dimension to uncover fatigue mechanisms. A study in 1,500 m freestyle athletes showed that miR-144-3p, miR-145-3p and miR-509-5p were significantly upregulated in circulating exosomes after fatigue exercise. Their target genes were enriched in vascular endothelial growth factor (VEGF) signaling pathway and glutathione metabolism. This change echoes the mechanism that miR-126 improves angiogenesis by regulating VCAM-1, suggesting that exosomal miRNA may participate in fatigue recovery through oxidative stress regulation and vascular function optimization (Lai et al., 2023). A separate study has demonstrated that it is possible to regulate a specific circulating miRNA (c-miRNA), with fluctuations in its expression correlating with exercise intensity. This observation reflects the body’s adaptive response to various synergistic loads imposed by different forms of physical activity (Safdar et al., 2009). Therefore, exercise-induced alterations in the miRNA expression profile not only enhance our understanding of the effects of exercise on the body but also establish a foundation for future research on exercise interventions.

There are notable differences in miRNA expression associated with various types of exercise, highlighting the complexity of muscle adaptation within the body under different exercise modalities. Research has demonstrated that at the molecular level, resistance training (such as strength training) and aerobic exercise (such as endurance training) are governed by distinct mechanisms involving miRNA regulation. For instance, a study comparing resistance training to aerobic exercise—specifically high-intensity interval training—provided evidence indicating that resistance training significantly modulates levels of miR-23a and miR-206 in muscle tissue compared to high-intensity interval training. This disparity may arise from the differing physiological stress responses elicited by various forms of exercise; resistance training typically results in greater muscle damage and subsequent repair processes, suggesting a potential positive feedback mechanism for specific miRNAs involved in muscle recovery. Moreover, cross-sectional studies examining the effects of exercise have identified gender as a significant factor influencing miRNA responses to different types of physical activity. There exist gender-specific variations in the regulation of miRNA expression post-exercise, which may be linked to underlying sex physiology (Safdar et al., 2009). Consequently, an in-depth investigation into how different types of exercise affect miRNA can not only provide a scientific foundation for developing enhanced intervention and rehabilitation strategies but also offer theoretical insights into preventing the onset and progression of exercise-induced fatigue.

### 3.4 Mechanism of miRNA in exercise fatigue

#### 3.4.1 Relationship between miRNA and energy metabolism

**FIGURE 3:**
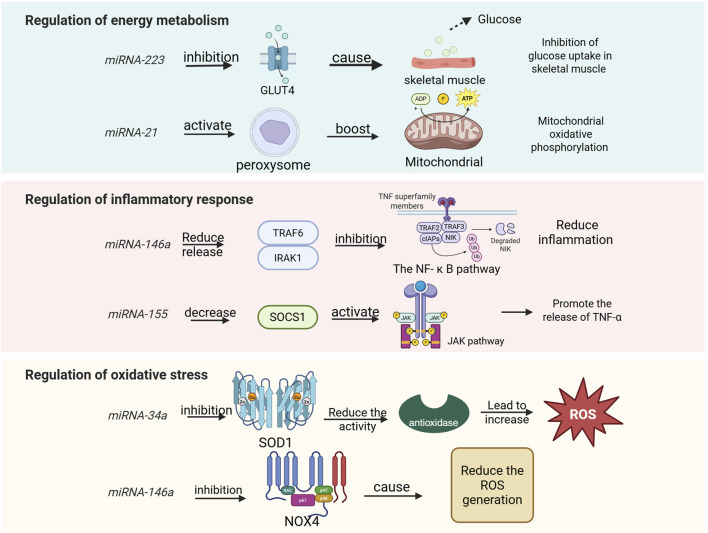
miRNAs involved in human regulation of energy metabolism, inflammatory responses, and oxidative stress.

As a type of non-coding RNA, miRNA has garnered significant attention for its role in the regulation of energy metabolism. miR-223 targets glucose transporter 4 (GLUT4) and inhibits its expression, thereby reducing glucose uptake in skeletal muscle (Li et al., 2025). Additionally, miR-223 was implicated in inflammation and the TLR4/NF-κB inflammatory pathway, which was important in exercise induced inflammatory response (M’baya-Moutoula et al., 2018). miR-375 regulates the function of islet β cells and influences insulin secretion; its abnormal expression is closely associated with insulin resistance (Meeusen et al., 2006). Exercise enhances skeletal muscle insulin sensitivity and ameliorates glucose metabolism disorders by upregulating the expression of the miR-29 family (Lu et al., 2010). The co-expression of miR-33 and sterol regulatory element-binding protein 2 (SREBP2) inhibits fatty acid β-oxidation while promoting cholesterol synthesis (Keller and Perez, 2022). Overexpression of miR-122 in the liver can decrease hepatic fat deposition by modulating lipid metabolism-related genes such as fatty acid synthase (FASN) and stearoyl-CoA desaturase 1 (SCD1) (Dalgaard et al., 2022). Furthermore, exercise promotes fatty acid oxidation and mitigates lipid accumulation through downregulation of miR-34a and activation of sirtuin 1 (SIRT1), a deacetylase (Horie et al., 2010). Under hypoxic conditions, upregulation of miR-210 inhibits mitochondrial respiratory chain complex activity while promoting glycolysis—a phenomenon known as the Warburg effect (Guo et al., 2023). Abnormal expression levels of miR-30a-5p may lead to disturbances in myocardial mitochondrial energy metabolism within models of pulmonary hypertensive right heart failure (Chen et al., 2019); however, exercise intervention could potentially restore mitochondrial function by regulating this specific miRNA. Moreover, certain miRNAs enriched in young plasma-derived small extracellular vesicles (sEVs), such as miR-21, activate peroxisome proliferator activated receptor gamma coactivator 1-alpha (PGC-1α), enhance mitochondrial oxidative phosphorylation, and alleviate age-related metabolic decline (Ismaeel et al., 2022). Collectively, these findings underscore that miRNAs play critical roles in regulating glucose metabolism, lipid metabolism, mitochondrial function, and oxidative metabolism (Figure 3). The CRF, which can be divided into “inflammation-driven” (high IL-6/miR-223), “leptin-related” (high leptin/miR-34a) and “depression-related type”, in which miR-223 exacerbates mitochondrial dysfunction by inhibiting PGC-1 α (Schmidt et al., 2024), illustrates the complementary mechanism of mitochondria inhibition of mitochondrial biosynthesis. This suggests mirna intervention strategies targeting different subtypes, such as miR-223 inhibitors for inflammatory type CRF.

Given the pivotal role of miRNAs in energy metabolism, and considering that exercise is a process characterized by high energy demand, the body regulates miRNA expression to maintain homeostasis in both energy metabolism and exercise regulation (Lv et al., 2024). Aerobic exercise significantly upregulates miR-1 and miR-133a, which promote glucose uptake and fatty acid oxidation in skeletal muscle (Harmon et al., 2017; Qian et al., 2024). In contrast, resistance exercise enhances the expression of miR-206, inhibiting genes associated with muscle atrophy (such as HDAC4) to sustain metabolic homeostasis within muscle tissue (Foley et al., 2006). Exercise also stimulates the release of exosomes from both muscle and adipose tissues; these exosomes carry specific miRNAs such as miR-30a-5p and miR-486 that can regulate the metabolism of distant target organs (Winbanks et al., 2013; Luu et al., 2020). For instance, exosomal miRNA derived from hypothalamic neural stem cells has been shown to improve systemic energy metabolism by inhibiting the TLR4/NF-κB inflammatory pathway (Huang et al., 2019). Furthermore, physical activity alters miRNA promoter activity through mechanisms involving DNA methylation and histone modification (Wu et al., 2022). The elevated levels of miR-34a induced by a high-fat diet can be reversed through exercise intervention, thereby restoring SIRT1-mediated metabolic balance (Chen et al., 2019). Additionally, miR-208a inhibits the PPARα signaling pathway, reducing the expression of fatty acid transporter CD36 (Tong et al., 2021). This inhibition impedes lipid utilization and forces reliance on anaerobic metabolism, resulting in lactic acid accumulation and subsequent exercise fatigue. Conversely, miR-27b activates the AMPK pathway to promote lipolysis and mitochondrial β-oxidation (Hu et al., 2023); this action enhances fat-derived energy supply efficiency while delaying energy depletion during prolonged physical activity. Studies have demonstrated that adipose-derived exosomal miR-124-3p inhibits hepatic lipid metabolism by targeting PPARγ, which leads to triglyceride accumulation and may indirectly exacerbate energy metabolism disorders during exercise (Zhao et al., 2021). Following high-intensity exercise, the expression patterns of certain miRNAs undergo significant changes. The upregulation of specific miRNAs, such as miR-144-3p and miR-145-3p, is associated with the stimulation of long-term potentiation (LTP) and signaling pathways related to vascular endothelial growth factor (VEGF) (You et al., 2016). Additionally, miR-146a-5p targets Table 1 to inhibit the NF-κB pathway, thereby reducing the release of inflammatory factors (Chen et al., 2022). This action protects mitochondria from oxidative damage and alleviates exercise-induced fatigue. Collectively, these studies suggest that miRNAs play a crucial role in regulating energy metabolism following exercise.

#### 3.4.2 The role of miRNA in inflammatory response

In recent years, an increasing number of studies have demonstrated that miRNAs play a crucial role in the regulation of inflammatory responses. These miRNAs can modulate the activation and inhibition of inflammatory signaling pathways by targeting genes associated with inflammation. For instance, miR-124 facilitates the polarization of macrophages towards the anti-inflammatory M2 phenotype by targeting C/EBP-α (Ponomarev et al., 2011). Similarly, miR-155 promotes the differentiation of Th1 cells while inhibiting the differentiation of Th2 cells through its action on c-Maf (Rodriguez et al., 2007). These findings indicate that miRNAs are integral to inflammatory responses by regulating inflammatory signaling pathways, mediators, and immune cell functions.

Exercise-induced fatigue can be categorized into central fatigue and peripheral fatigue, with the latter being closely associated with muscle damage and inflammatory responses. High-intensity or prolonged exercise can trigger an inflammatory response in muscle tissue, characterized by the release of inflammatory factors (such as IL-6 and TNF-α) and the infiltration of immune cells. This inflammatory response is not only a necessary process for the body to repair damage but may also exacerbate fatigue and delay recovery. For instance, NF-κB serves as a central transcription factor in the inflammatory response. miR-146a negatively regulates the NF-κB signaling pathway and inhibits inflammation by targeting TRAF6 and IRAK1 (Taganov et al., 2006). The upregulation of miR-146a may mitigate inflammation-mediated muscle damage during exercise-induced fatigue. Similarly, miR-21 reduces activation of the MAPK signaling pathway by targeting PDCD4, thereby diminishing the inflammatory response (Sheedy et al., 2010). Additionally, miR-206 inhibits inflammation through its action on IL-6 (McCarthy et al., 2007). In cases of exercise-induced fatigue, increased expression of miR-206 and miR-210 may alleviate symptoms by reducing the release of pro-inflammatory factors. These microRNAs have potential therapeutic roles in addressing exercise-related fatigue by modulating either inflammatory responses or the secretion of inflammatory mediators. However, it is important to note that certain miRNAs can also contribute to exacerbating exercise-induced fatigue via their influence on inflammation. The miR-155 enhances the JAK/STAT signaling pathway and promotes the production of inflammatory factors by targeting SOCS1 (O’Connell et al., 2007). In the context of exercise-induced fatigue, the upregulation of miR-155 expression may exacerbate the inflammatory response, thereby contributing to increased fatigue. Conversely, miR-126 inhibits monocyte migration to sites of inflammation by targeting VCAM-1 (Harris et al., 2008). During exercise fatigue, elevated levels of miR-126 may mitigate inflammation-mediated muscle damage through a reduction in immune cell infiltration. Additionally, miR-34a elevates oxidative stress and inflammation by targeting SIRT1(Yamakuchi et al., 2008). In cases of exercise fatigue, an increase in miR-34a expression could worsen oxidative stress and inflammatory responses, further intensifying feelings of fatigue. It is worth noting that miRNA also shows the potential for cross-system regulation in neurological disease-related fatigue. For example, in multiple sclerosis (MS), miR-126 suppresses immune cell infiltration into the CNS by targeting vascular cell adhesion molecule 1 (VCAM-1), thereby reducing neuroinflammation-mediated fatigue. A study based on wearable sensors showed that fatigue in ms patients was positively correlated with sympathetic activity (e.g., reduced heart rate variability), but negatively with miR-126 expression level. This mechanism is highly similar to the role of mir-126 in alleviating muscle inflammatory injury by inhibiting vcam-1 during exercise fatigue (Moebus et al., 2024) In post-infection ME or CFS patients, HHV-6 viral load was positively correlated with mir-155 expression, which promotes pro-inflammatory factor (e.g., TNF- α) release through activation of the JAK or STAT pathway (Gravelsina et al., 2022). Also confirmed the mechanism by which miR-155 enhances the inflammatory response by targeting socs1, suggesting that miR-155 can be used as an early warning marker for fatigue associated with viral infection. Moreover, miR-21 is found to be upregulated in patients with chronic fatigue syndrome (CFS) and facilitates inflammatory responses by targeting PDCD4 (Bjersing et al., 2015). Both miR-1 and miR-133 are also upregulated following muscle injury due to exercise; they play crucial roles in regulating muscle repair processes by targeting genes associated with inflammation (Chen et al., 2006).

Overall, miRNA significantly influence exercise-related fatigue through their regulation of inflammatory signaling pathways and mediators. Abnormal expression patterns of these miRNA are closely linked to both the onset and progression of exercise-induced fatigue. Investigating the specific regulatory mechanisms employed by miRNA within the context of inflammation during exercise-induced fatigue presents a promising avenue for research within sports medicine and rehabilitation.

#### 3.4.3 Interaction between miRNA and oxidative stress

**FIGURE 4:**
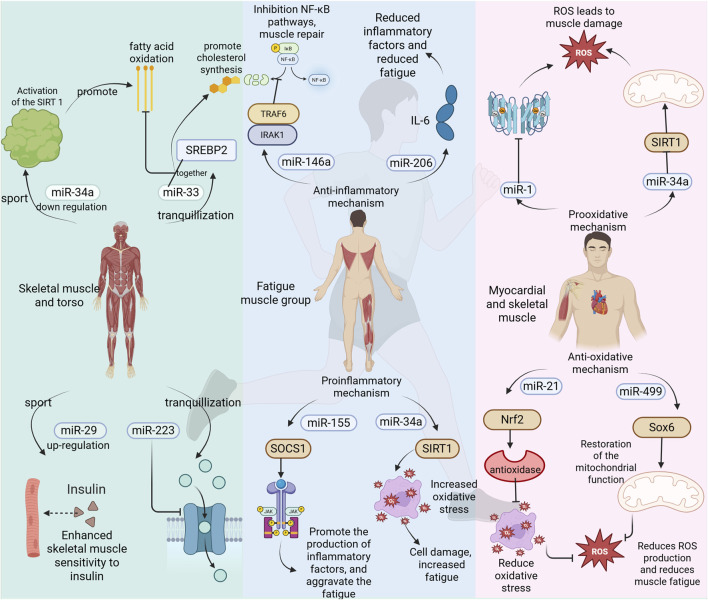
miRNAs involved in the regulation of exercise fatigue.

Oxidative stress is a condition characterized by an imbalance between the production and clearance of intracellular reactive oxygen species (ROS), which is closely associated with the onset and progression of various diseases, including cardiovascular diseases, neurodegenerative disorders, and cancer. The miRNAs play a crucial role in oxidative stress by modulating the expression of genes related to oxidative stress and influencing both the production and elimination of ROS. For instance, miR-34a and miR-1 are implicated in regulating ROS production, while miR-146a and miR-21 are involved in ROS clearance (Figure 4). Additionally, miR-133a and miR-499 regulate mitochondrial function. Furthermore, miRNAs significantly contribute to oxidative stress-related diseases. The miR-210 inhibits mitochondrial function, increases ROS production, and exacerbates myocardial ischemia-reperfusion injury by targeting ISCU (Chan et al., 2009). Similarly, miR-155 impairs the antioxidant defense system, elevates oxidative stress levels, and facilitates the progression of Alzheimer’s disease through its interaction with Nrf2 (Guedes et al., 2014). By targeting ZEB1, miR-200c diminishes the activity of antioxidant enzymes, enhances ROS generation, and promotes apoptosis in cancer cells (Magenta et al., 2011). These studies underscore the pivotal role that miRNAs play in oxidative stress as well as their underlying mechanisms; moreover, abnormal expression patterns of these molecules are closely linked to the development and advancement of various diseases.

During exercise, particularly during high-intensity or prolonged activities, the activity of the mitochondrial electron transport chain is enhanced, resulting in an excessive production of ROS. When ROS levels exceed the body’s antioxidant capacity, oxidative stress is triggered. This can lead to lipid peroxidation, protein oxidation, and DNA damage within cell membranes, ultimately causing muscle fatigue and functional decline. The miRNAs play a crucial role in regulating both the production and clearance of ROS by targeting genes associated with oxidative stress; thus, they influence the onset and progression of exercise-induced fatigue. There are conflicting reports regarding the impact of miRNAs on exercise fatigue. The following miRNAs may exacerbate its occurrence and development: miR-34a targets SIRT1 and inhibits its expression, leading to mitochondrial dysfunction and an overproduction of reactive oxygen species (ROS) (Yamakuchi et al., 2008). In the context of exercise-induced fatigue, the upregulation of miR-34a expression may exacerbate oxidative stress and worsen feelings of fatigue. Similarly, miR-1 reduces the activity of antioxidant enzymes by targeting superoxide dismutase 1 (SOD1) and superoxide dismutase 2 (SOD2), thereby increasing ROS production (Chen et al., 2006). During episodes of exercise-related fatigue, elevated levels of miR-1 may further intensify oxidative stress, resulting in muscle damage and heightened sensations of fatigue. Furthermore, miR-133a impairs mitochondrial biosynthesis and function through its action on peroxisome proliferator-activated receptor gamma coactivator 1-alpha (PGC-1α), contributing to excessive ROS generation (Safdar et al., 2009). In scenarios involving exercise-induced fatigue, increased expression levels of miR-133a could aggravate both mitochondrial dysfunction and oxidative stress. The following miRNA may mitigate the onset and progression of exercise-induced fatigue. miR-146a inhibits NADPH oxidase activity and reduces reactive oxygen species (ROS) production by targeting NOX4 (Cheng et al., 2013). In the context of exercise fatigue, upregulation of miR-146a expression may alleviate oxidative stress and diminish fatigue symptoms. Notably, miRNA exhibits a similar neural-oxidative stress-inflammation interaction regulatory mechanism in neurodegenerative diseases (e.g., Alzheimer’s disease AD, Parkinson’s disease PD). For example, miR-124-3p relieves neuronal damage in the ad model by targeting the MAPK pathway to inhibit microglial activation, reduce ROS generation and the release of neuroinflammatory factors (e.g., IL-6), while mir-132 enhances mitochondrial antioxidant enzyme activity by regulating BDNF expression and improves dopaminergic neuronal function in the PD model (Azam et al., 2024). Similarly, miR-21 enhances the Nrf2 signaling pathway and promotes the expression of antioxidant enzymes by targeting PDCD4 (Rodriguez et al., 2007). During episodes of exercise fatigue, increased levels of miR-21 may help reduce oxidative stress through bolstering the antioxidant defense system. Furthermore, miR-499 facilitates mitochondrial function recovery while decreasing ROS production by targeting Sox6 (Wang et al., 2011) (Figure 4). In cases of exercise fatigue, elevated expression of miR-499 could enhance mitochondrial functionality and lower oxidative stress. Additionally, miRNAs play a significant role in diseases associated with exercise-related fatigue. For instance, in patients with chronic fatigue syndrome, downregulation of miR-16 leads to inhibited activity of antioxidant enzymes and heightened oxidative stress via its target SOD2 (Bjersing et al., 2015). Conversely, after muscle injury induced by exercise, there is an upregulation of miR-206 which mitigates inflammation and oxidative stress through its action on IL-6 (Sheedy et al., 2010). Oxidative stress represents one critical mechanism underlying exercise-induced fatigue; it arises from excessive ROS production coupled with an imbalance in the antioxidant defense system. Thus, miRNA plays a pivotal role in regulating both ROS generation and clearance as well as maintaining the integrity of the antioxidant defense system; any aberration in their expression is closely linked to the emergence and progression of exercise-related fatigue.

### 3.5 Future research directions and clinical applications

As a class of small non-coding RNA molecules, miRNAs play a crucial role in the life processes of eukaryotes. miRNAs can inhibit the translation and degradation of target mRNA by binding to it, and they can also influence gene expression through the regulation of DNA methylation and histone modification. Consequently, miRNAs are integral to various cellular functions including proliferation, differentiation, apoptosis, metabolism, and stress responses. Moreover, miRNAs regulate the onset and progression of exercise-induced fatigue by modulating oxidative stress, inflammatory responses, energy metabolism, and neuroendocrine pathways. For instance, miR-34a targets SIRT1 to regulate cellular oxidative stress by inhibiting mitochondrial function and increasing reactive oxygen species (ROS) production (Yamakuchi et al., 2008). Similarly, miR-146a inhibits the NF-κB signaling pathway by targeting TRAF6 and IRAK1, thereby reducing inflammation (Taganov et al., 2006). Additionally, miR-133a regulates energy metabolism through its action on PGC-1α while inhibiting mitochondrial biosynthesis (Safdar et al., 2009). These specific miRNAs are pivotal regulators in the context of exercise fatigue. To further elucidate the functional mechanisms underlying these effects of miRNA on exercise fatigue, high-throughput sequencing combined with bioinformatics analysis will be employed to identify relevant miRNAs along with their target genes associated with this condition. Furthermore, verification of their functional mechanisms is essential. The intricate regulatory network involving miRNA in relation to oxidative stress response as well as inflammatory reactions warrants additional investigation. In terms of epigenetic regulation mechanisms related to exercise fatigue mediated by miRNA continue to be explored actively. Moreover, attention has been directed towards understanding how factors such as exercise intensity, duration, and type impact the expression levels of specific miRNAs. miRNA only one class of small non-coding RNA molecules, there are other type non-coding RNA play a important role in exercise. Such as long non-coding RNAs (lncRNAs), which transcripts >200 nucleotides with no protein-coding potential in mediating the body’s response to exercise. These molecules regulate gene expression epigenetically, transcriptionally, and post-transcriptionally, influencing metabolic, cardiovascular, and musculoskeletal adaptations.

It is of significant clinical importance to investigate the role of miRNA in exercise-induced fatigue. miRNA has the potential to serve as a diagnostic biomarker for exercise fatigue, allowing for the assessment of both the severity and recovery from such fatigue through the analysis of miRNA expression profiles in blood, urine, or saliva. Various combinations of miRNAs have been screened to enhance the accuracy and specificity of diagnosing exercise-related fatigue. Moreover, miRNAs can be targeted therapeutically for managing exercise fatigue; specific agonists (e.g., miR-30e agonists) and inhibitors (e.g., miR-34a inhibitors) have been developed to modulate signaling pathways associated with exercise fatigue. Utilizing CRISPR/Cas9 technology enables precise editing of miRNA genes, rendering them effective targets for addressing exercise-induced fatigue. Gene therapy using adeno-associated virus (AAV) vectors has emerged as a leading approach for delivering therapeutic genes, while microRNAs (miRNAs) offer precise gene regulation. Combining these technologies enables targeted, durable treatments for exercise related diseases (Wang, et al., 2019). Personalized exercise regimens may be formulated based on individual miRNA profiles, facilitating tailored training programs that optimize performance while minimizing feelings of exhaustion post-exercise. Additionally, research has explored the involvement of miRNAs in sports nutrition interventions—specifically regarding antioxidants and anti-inflammatory agents. The investigation into the role of miRNAs in exercise-related fatigue offers novel insights into both diagnosis and treatment strategies. Future research directions should focus on elucidating functional mechanisms, regulatory processes governing expression levels, and individual variability among different subjects concerning their respective miRNA profiles. In clinical practice, leveraging miRNAs as diagnostic markers and therapeutic targets presents a promising strategy for achieving.

## 4 Conclusions and outlook

Studies in the field of sports fatigue have increasingly focused on the circulation features of miRNA, thereby further confirming its significant biological value. Current literature indicates that miRNA is involved in regulating various cellular processes, including energy metabolism, inflammatory responses, and oxidative stress. These mechanisms not only provide an objective explanation for the physiological causes of exercise-induced fatigue but also open new avenues for understanding this concept.

The findings from recent studies demonstrate a correlation between miRNA levels and exercise fatigue. This suggests that different types of miRNAs may serve distinct regulatory functions across various forms of exercise-related fatigue; therefore, when investigating biomarkers associated with exercise fatigue, it is essential to consider these diverse aspects and relevant findings in a coordinated manner. Future research should focus on establishing causal relationships between miRNA and exertion, which is crucial for comprehending the specific roles this molecule plays in regulating adaptation and recovery processes related to exercise. In a similar vein, the challenges associated with the potential of miRNA as a biomarker should not be underestimated. The analysis of specific miRNAs in human serum has the capacity to predict both the degree of exercise-induced fatigue and recovery status. This provides a scientific foundation for athlete training and rehabilitation, facilitating the development of individualized training programs that enhance athletic performance and mitigate sports injuries. However, it is crucial to acknowledge that while the properties of miRNA appear advantageous for investigating exercise fatigue, further empirical research is necessary to validate its prospects in clinical practice. Continued emphasis on scientific inquiry into the physiological functions of miRNA related to exercise is essential, as this will help expand its application potential and future developments concerning exercise fatigue.

Autophagy, a critical cellular recycling process, has emerged as a key mechanism in exercise-induced fatigue regulation. During prolonged exercise, autophagy clears damaged organelles (e.g., dysfunctional mitochondria) and protein aggregates, maintaining cellular homeostasis. miRNAs intricately regulate autophagy pathways: for instance, miR-30e suppresses autophagy initiation by targeting *BECN1* (beclin-1), while miR-223 enhances autophagic flux via *FOXO3* modulation. Impairments in exercise-triggered autophagy exacerbate oxidative stress and energy depletion, accelerating fatigue onset. Notably, endurance training upregulates miR-30e, promoting mitochondrial biogenesis through PGC-1α activation while fine-tuning autophagic activity to balance clearance and conservation of cellular resources. Future studies should explore miRNA-mediated autophagy as a therapeutic target to delay fatigue and enhance recovery (Safdar et al., 2016). The gut-muscle axis represents a novel frontier in exercise fatigue research. Gut microbiota dysbiosis during intense exercise impairs intestinal barrier integrity, increasing systemic inflammation and oxidative stress. miRNAs modulate this axis: miR-146a downregulates TLR4/NF-κB signaling in gut epithelial cells, reducing inflammation-induced fatigue, while miR-21 targets *PDCD4* to maintain mucosal homeostasis. Conversely, microbial metabolites (e.g., short-chain fatty acids) influence host miRNA expression—butyrate upregulates *miR-200c*, enhancing mitochondrial function in skeletal muscle. Exercise-induced shifts in *Firmicutes/Bacteroidetes* ratio correlate with circulating miR-486 levels, suggesting microbiota-driven miRNA regulation of energy metabolism. Harnessing this crosstalk via probiotics or miRNA-targeted interventions may mitigate fatigue (Li et al., 2025).

In conclusion, a deeper understanding of the functions and regulatory mechanisms governing miRNAs will provide more robust theoretical support and effective references for advancing sports medicine.
